# Improved stability and activity of laccase through *de novo* and post-synthesis immobilization on a hierarchically porous metal–organic framework (ZIF-8)†

**DOI:** 10.1039/d3ra01571h

**Published:** 2023-06-08

**Authors:** Ran Xu, Xujie Zhang, Osman Ahmend Zelekew, Eduardo Schott, Yi-nan Wu

**Affiliations:** a College of Environmental Science and Engineering, State Key Laboratory of Pollution Control and Resource Reuse, Tongji University 1239 Siping Rd. Shanghai 200092 China 51n@tongji.edu.cn; b Shanghai Institute of Pollution Control and Ecological Security 1239 Siping Rd. Shanghai 200092 China; c Department of Materials Science and Engineering, Adama Science and Technology University Adama Ethiopia; d Department of Inorganic Chemistry of the Faculty of Chemistry and Pharmacy, Pontificia Universidad Católica de Chile Vicuña Mackenna 4860, Macul Santiago Chile edschott@uc.cl

## Abstract

Porous materials such as metal–organic frameworks (MOFs) are considered to be suitable materials for immobilizing enzymes to improve their stability. However, conventional MOFs reduce the enzymes' catalytic activity due to difficulties with mass transfer and diffusing reactants after their micropores are occupied by enzyme molecules. To address these issues, a novel hierarchically structured zeolitic imidazolate framework-8 (HZIF-8) was prepared to study the effects of different laccase immobilization approaches such as the post-synthesis (LAC@HZIF-8-P) and *de novo* (LAC@HZIF-8-D) immobilization of catalytic activities for removing 2,4-dichlorophenol (2,4-DCP). The results showed higher catalytic activity for the laccase-immobilized LAC@HZIF-8 prepared using different methods than for the LAC@MZIF-8 sample, with 80% of 2,4-DCP removed under optimal conditions. These results could be attributable to the multistage structure of HZIF-8. The LAC@HZIF-8-D sample was stable and superior to LAC@HZIF-8-P, maintaining a 2,4-DCP removal efficiency of 80% after three recycles and demonstrating superior laccase thermostability and storage stability. Moreover, after loading with copper nanoparticles, the LAC@HZIF-8-D approach exhibited a 2,4-DCP removal efficiency of 95%, a promising finding for its potential use in environmental purification.

## Abstract 2

Porous materials such as metal–organic frameworks (MOFs) are considered to be suitable materials for immobilizing enzymes to improve their stability.

## Graphical abstract

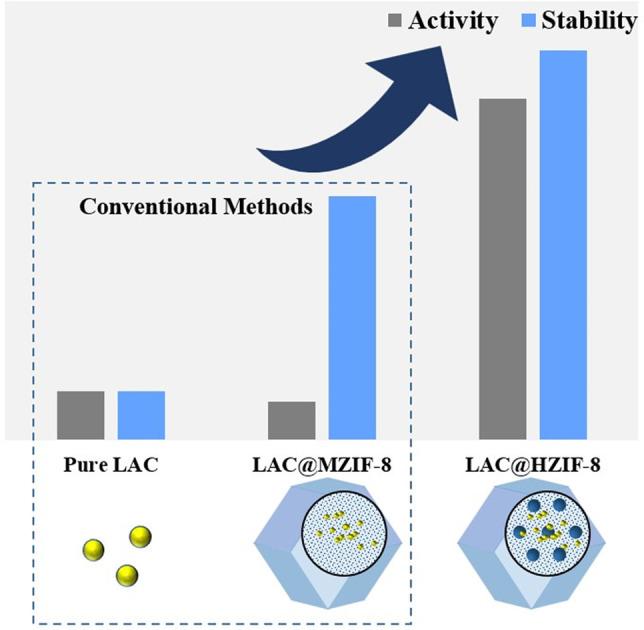

## 1 Introduction

Phenolic compounds are widely used in the industrial production of wood preservatives, pigments, herbicides, and pesticides.^1^ As populations expand and industries expand, these compounds are constantly released into the aquatic environment, where they exert harmful effects on the ecological system.^2^ Among the harmful phenolic derivatives, chlorophenol compounds are the most extensively reported since they pollute water at levels of only 1 mg L^−1^, with severe implications for public health.^3^ To date, the success of chemical and physical approaches for treating toxic phenolic compounds has been limited^4^ by the pollution caused by secondary by-products and the high costs involved.^5^ Hence, research has refocused on biological methods as low-cost, low-polluting means of removing chlorophenols from wastewater.^6^

Biological catalysis provides an environmentally-friendly option due to its lower energy requirements, easier operating conditions, and non-toxic products.^7^ Among enzymes, laccase has been used extensively to remove chlorophenols from wastewater.^8^ However, free laccase has low thermal stability, a narrow pH application range, weak organic solvent tolerance, and a low reuse rate, all of which limit its application in industry.^9–12^ Accordingly, enzyme immobilization technologies using insoluble carriers are required to confine enzymes to a particular space. However, current immobilized enzyme carriers exhibit shortcomings such as uncontrollable pore size, high preparation costs, enzyme leaching, poor product stability, and reduced enzyme activity.^13–15^ However, metal–organic frameworks (MOFs) have attracted greater attention due to their high specific surface area and pore volume, their designable and controllable structure, and their chemical and thermal stability.^16–19^

Depending on the sequence used, the strategies for synthesizing MOF-immobilized enzymes consist of *de novo* encapsulation and post-synthetic packaging.^20^ For example, one research group describes *biomineralization*, which is widely used in *de novo* encapsulation.^21–23^*Co-precipitation* and *mechanochemical encapsulation* have also demonstrated their efficiency for the *in situ* immobilization of enzymes.^24–26^ Similarly, post-synthetic packaging prepared with different kinds of MOFs has also garnered considerable attention for its ability to protect laccase.^27^ Yet MOFs are mostly microporous, which is not conducive to diffusion and mass transfer; a *mesoporous* or graded pore structure matched to the enzyme size would be ideal. For this reason, hierarchical MOFs can not only protect the enzyme from external stimulation but also selectively transport the substrate and reaction products.^28^ These considerations motivated the present study's design of *in situ* and post-synthesis enzyme immobilization strategies.

Accordingly, a microporous and hierarchical ZIF-8 (MZIF-8/HZIF-8) was prepared for the study, with the HZIF-8 immobilized enzyme catalysts also developed *via in situ* and post-synthesis methods in the aqueous phase. These materials were then evaluated for their ability to remove 2,4-DCP. The removal efficiency of the LAC@HZIF-8 was predicted to exceed that of the LAC@MZIF-8 and LAC samples. In addition, the de novo-synthesized LAC@HZIF-8 sample (LAC@HZIF-8-D) was also expected to display greater stability than the MZIF-8 and post-synthesis (LAC@HZIF-8-P) samples. These investigations aimed to identify which enzyme immobilization strategy offers greater environmental stability and reuse ability, thereby improving the industrial application of laccase.

## 2 Experimental

### 2.1 Chemicals and reagents

The laccase and 2,4-DCP were obtained from Sigma-Aldrich. The Zn(NO_3_)_2_·6H_2_O, triethylamine, l-histidine, and cetyltrimethylammonium bromide were provided by Hushi Co. (Shanghai, China) and the 2-methylimidazole was purchased from OKA Co. (Beijing, China). All other chemicals were of the highest commercially available grade and were used as received.

### 2.2 Methods

#### 2.2.1 Synthesis of HZIF-8 and MZIF-8

The HZIF-8 was prepared according to our previous work.^29^ In this procedure, Zn(NO_3_)_2_·6H_2_O (1 mmol) was first dissolved in deionized water (25 mL), and appropriate amounts of l-histidine (His) and cetyltrimethylammonium bromide (CTAB) with a 4 : 1 molar ratio were added and dispersed ultrasonically. 2-Methylimidazole (8 mmol) was then dissolved in deionized water (25 mL), and triethylamine (8 mmol) was added to the resulting solution. The two solutions were mixed and placed on a magnetic stirrer for 1 h at room temperature. The resulting product was centrifuged at 10 000 rpm, washed with a mixture of ethanol and water (1 : 1), and freeze-dried overnight to produce HZIF-8, a white powder.

The MZIF-8 was also prepared in line with previous research.^30^ First, Zn(NO_3_)_2_·6H_2_O (1 mmol) was dissolved in deionized water (25 mL). Subsequently, 2-methylimidazole (8 mmol) was dissolved in deionized water (25 mL), and triethylamine (8 mmol) was added to the solution. The Zn(NO_3_)_2_·6H_2_O and 2-methylimidazole solutions were then mixed and placed on a magnetic stirrer for 1 h at room temperature. After centrifugation and freeze-drying overnight, the white MZIF-8 powder was obtained.

#### 2.2.2 Synthesis of LAC@HZIF-8-D

The *de novo* synthesis of the LAC@ZIF-8-D MOF was performed as follows: first, a certain quantity of laccase was dissolved in a pH 5 citric acid–disodium phosphate (CPBS) buffer solution (1 mL). This was then added to a Zn(NO_3_)_2_·6H_2_O solution under ultrasonication, with the procedure then following that of the HZIF-8 preparation process described above.

#### 2.2.3 Synthesis of LAC@HZIF-8-P

The LAC@HZIF-8-P was prepared as follows: a certain amount of HZIF-8 was first dissolved in 50 mL of deionized water and the laccase was then dissolved in 1 mL of a pH 5 citric acid–disodium phosphate (CPBS) buffer solution and added to the HZIF-8 solution. This mixture was then stirred for 1 h, centrifuged, and freeze-dried overnight, producing the white LAC@HZIF-8-P powder. The products were stored in a refrigerator at 4 °C to prevent the immobilized enzyme from being inactivated.

#### 2.2.4 Synthesis of nCu/LAC@HZIF-8

The copper nanoparticles were synthesized according to previous research^31^ and loaded onto the LAC@ZIF-8 as follows. First, 25 mg of laccase was dissolved in 1 mL of citrate disodium hydrogen phosphate (CPBS) buffer solution (pH 5). Next, 25 mg of the copper nanoparticles was dispersed in 1 mL of deionized water. The laccase and copper nanoparticle solutions were then added to a solution containing Zn(NO_3_)_2_·6H_2_O, followed by ultrasonication. The synthesis then followed the procedure for the HZIF-8 immobilized enzyme described above to produce nCu/LAC@HZIF-8. After freeze-drying, the product was ground and stored in a refrigerator at 4 °C.

#### 2.2.5 Laccase activity assay

The laccase activity assay followed the procedure described in Zhang *et al.*^32^ using 2,4-DCP as a substrate. The free laccase (1 mg) or LAC@ZIF-8 samples containing the same amounts of enzyme were then added to the citric acid–disodium hydrogen phosphate (CPBS) buffer solution (pH 7) with ultrasonic dispersion. This solution was then added to the 2,4-DCP solution (20 mg L^−1^, 25 mL) and stirred to produce the reaction. After a certain reaction time, the absorbance of the solution was measured at 510 nm. The rate of 2,4-DCP removal was calculated according to eqn (1):1
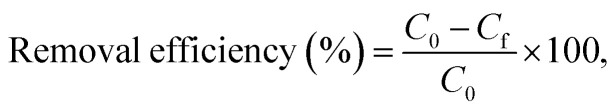
where *C*_0_ and *C*_f_ are the initial and final concentrations (mg L^−1^) of 2,4-DCP in the solution, respectively.

The effects of the different reaction times were determined based on laccase activity. The absorbance of the solutions was measured at 15 min, 30 min, 1 h, 2 h, 4 h, and 6 h after the reaction without or after centrifugation for free laccase or the LAC@ZIF-8 sample, respectively. The temperature tolerance of the LAC@ZIF-8 and free laccase was determined by evaluating their enzymatic activity at 20, 30, 40, 50, 60, 70, and 80 °C, at specified pH levels (2, 3, 4, 5, 6, 7, 8 and 9) at each time interval. The storage ability of each sample was tested by detecting their enzymatic activity at 4 °C after 0, 3, 7, and 15 days.

### 2.3 Characterizations

The scanning electron microscopy (SEM) measurement was conducted using a ZEISS Gemini 300 microscope and a Hitachi 7700 transmission electron microscope was used to carry out the transmission electron microscopy (TEM) analysis. The X-ray diffraction (XRD) analysis was performed with a wide-angle X-ray diffractometer (D8 ADVANCE). The thermogravimetric analysis (TGA) was recorded on a Q600 SDT thermogravimetric analyzer at a detection range of 50–800 °C and a heating rate of 10 °C min^−1^. Fourier transform infrared spectra (FT-IR) were conducted on a Thermo Scientific Nicolet iS5 FT-IR spectrometer using KBr pellets in the range of 4000–500 cm^−1^. The confocal laser scanning microscopic (CLSM) analysis was performed using an Olympus FV1200 instrument to produce the fluorescence images of the immobilized enzyme. Nitrogen adsorption and desorption isotherms were recorded on an ASAP 2020 system and the surface areas were calculated using the Brunauer–Emmett–Teller (BET) equation.

## 3 Results and discussion

### 3.1 Characterizations

**Fig. 1:**
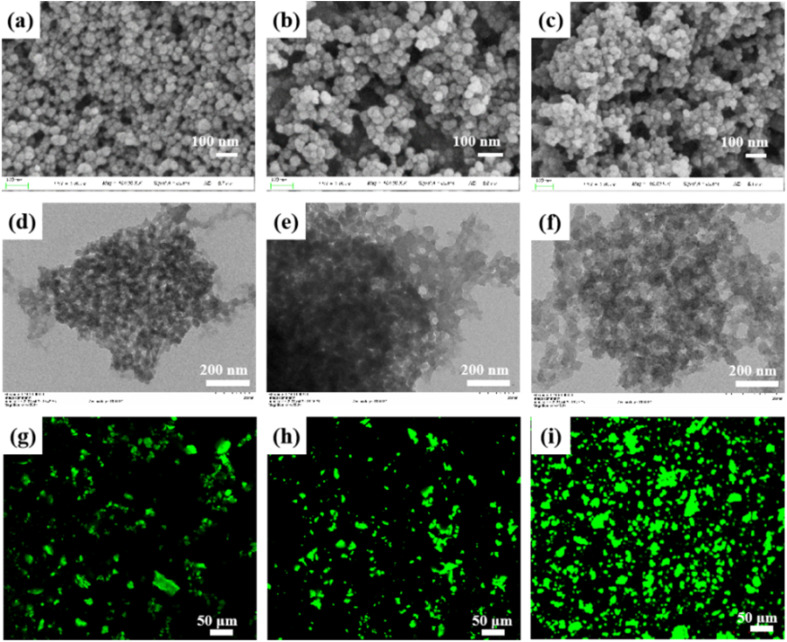
SEM images of (a) HZIF-8, (b) LAC@HZIF-8-D, and (c) LAC@HZIF-8-P. TEM images of (d) HZIF-8, (e) LAC@HZIF-8-D, and (f) LAC@HZIF-8-P. CLSM images of (g) LAC@MZIF-8, (h) LAC@HZIF-8-D, and (i) LAC@HZIF-8-P.

The morphologies of the prepared samples were examined and found to be similar. Fig. 1a–c indicate the SEM images of the HZIF-8, LAC@HZIF-8-D, and LAC@HZIF-8-P samples, respectively. Fig. 1d–f show the TEM images of the HZIF-8, LAC@HZIF-8-D, and LAC@HZIF-8-P samples, respectively. The dark areas in the TEM images (Fig. 1e and f) of the LAC@HZIF-8-D and LAC@HZIF-8-P samples differed from the HZIF-8 sample due to the agglomeration of the laccase blocked by the internal pores of the HZIF-8 synthesized *in situ*. However, the image of LAC@HZIF-8-P (Fig. 1f) shows fewer dark and white areas between the crystals, with the mesoporous HZIF-8 filled by laccase. Interestingly, the LAC@MZIF-8 (Fig. S1†) displayed a regular rhomboid dodecahedron shape and a more uniform crystal distribution, while the crystal structure of the synthesized MZIF-8 did not—a finding compatible with Gross's work.^33^ This difference may be attributed to the added laccase providing more growth sites and promoting crystal growth in the MZIF-8 sample.

Fig. 1g–i present the confocal laser microscopy images (CLSMs) of the LAC@MZIF-8 and LAC@HZIF-8 samples. Because the ZIF-8 is not fluorescent, the fluorescence signal originates from the FITC-labelled laccase. Fig. 1g depicts a sizeable uneven area of the fluorescent green substances in the MZIF-8 immobilized enzyme material. Because the particle size of HZIF-8 pore size does not closely match the large-size laccase, the laccase loading can be attributed primarily to the HZIF-8 mesopores rather than its micropores. The highly dispersed fluorescent signal depicted in Fig. 1h demonstrates the existence and uniform distribution of laccase across the entire HZIF-8 framework. The most dispersed signal was emitted from the LAC@HZIF-8-P, confirming it could hold the largest amount of the enzyme.

**Fig. 2:**
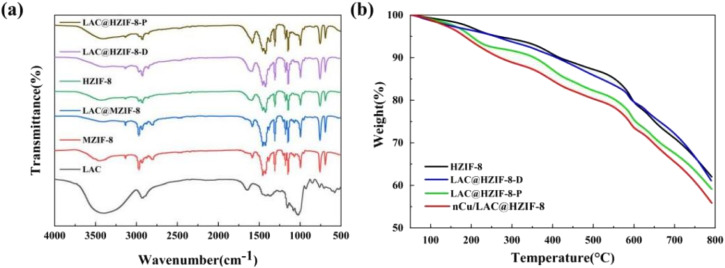
(a) The FT-IR patterns of MZIF-8, HZIF-8, LAC@MZIF-8, LAC@HZIF-8-D, and LAC@HZIF-8-P. (b) The TGA curves of the HZIF-8, LAC@HZIF-8-D, LAC@HZIF-8-P, and nCu/LAC@HZIF-8 samples.

The Fourier Transform Infrared Spectrometer (FT-IR) analysis of the samples confirmed the formation of ZIF-8 and the loading of laccase. The laccase FTIR spectrum (Fig. 2a) indicates an absorption peak of 1645 cm^−1^ representing the N–H and C–N stretching bands of the amide group in the laccase.^34^ Moreover, the typical absorption peaks of the HZIF-8 at 3132, 2929, 1681, 1596, 1147, 995, and 424 cm^−1^ were retained in LAC@HZIF-8, indicating that the addition of laccase did not affect the synthesis of HZIF-8.^35^ Note that the interaction between histidine in the laccase and the histidine template in the HZIF-8 synthesis system caused the absorption peak of the LAC@HZIF-8 to deviate. At its 1645 cm^−1^ peak, the FT-IR spectrum of the LAC@HZIF-8-P revealed an obvious shoulder peak relative to HZIF-8 and MZIF-8, and a slight shift of the absorption peak at 1596 cm^−1^ to 1582 cm^−1^. In contrast, the 1645 cm^−1^ peak of the LAC@MZIF-8 exhibited a slight shoulder peak relative to the MZIF-8, possibly due to the latter's unstable structure and poor crystallinity, which produced a lower laccase loading rate in the LAC@MZIF-8 sample.^36^ The electrophoresis performed under enzyme denaturant conditions using sodium dodecyl sulfate–polyacrylamide gel electrophoresis (SDS–PAGE) was also tested to confirm the presence of laccase in HZIF-8 (Fig. S4†). In general, both the LAC@HZIF-8-D and LAC@HZIF-8-P gels emitted a signal of 60 kDa, consistent with that emitted by the free laccase.

The weight lost from the samples was also measured. The weight of the laccase decreased rapidly below 300 °C while the MZIF-8 sample lost approximately 15% of its weight below 400 °C (Fig. S2†). However, as anticipated, the HZIF-8 synthesized using CTAB and histidine had a smaller weight loss ratio of about 9% in the same temperature range, which may be attributed to HZIF-8's higher specific surface area (Fig. 2b). The LAC@HZIF-8-P lost more weight than the HZIF-8 and LAC@HZIF-8 samples, which suggested its laccase loading rate was higher.^37^Fig. 2b shows, moreover, that the TG curve trend exhibited by the nCu/LAC@HZIF-8-D was consistent with that of the HZIF-8 and LAC@HZIF-8-D, with no noticeable weight loss stage. At 300 °C, the LAC@HZIF-8-D and nCu/LAC@HZIF-8-D lost 8.36% and 11.1% of their weight, respectively, suggesting that the Cu NPs occupied and were loaded onto the mesoporous HZIF-8.

**Fig. 3:**
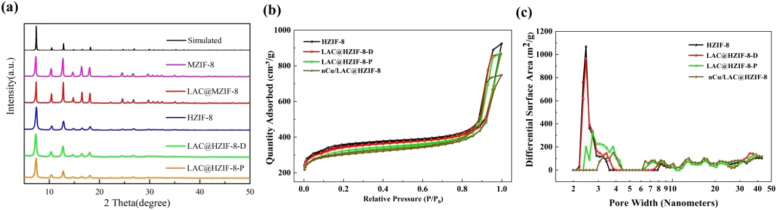
(a) The XRD pattern of the ZIF-8 and LAC@ZIF-8 samples. (b) N_2_ adsorption/desorption isotherms image of the HZIF-8, LAC@HZIF-8-D, LAC@HZIF-8-P, and nCu/LAC@HZIF-8 samples. (c) Pore size distribution image of the HZIF-8, LAC@HZIF-8-D, LAC@HZIF-8-P, and nCu/LAC@HZIF-8 samples.

Fig. 3a shows the XRD patterns of the MZIF-8, HZIF-8, and LAC@HZIF-8 samples. The former two samples demonstrated six characteristic diffraction peaks at 7.3°, 10.5°, 12.7°, 14.7°, 16.5°, and 18.3°, consistent with the results of tests stimulating ZIF-8 (ref. 38 and 39) and confirming the expected structure of the LAC@ZIF-8 crystals.

Moreover, the LAC@ZIF-8 patterns exhibited no impurity peak but produced a sharp peak indicating its high crystallinity and stable structure. Given that the catalyst activity performed more effectively with less space between the copper nanoparticles (Cu NPs) and laccase, we synthesized the nCu/LAC@HZIF-8 based on the synthesis of LAC@HZIF-8-D. As Fig. S5† indicates, the loading of Cu nanoparticles affected neither the structure of the crystals nor the LAC@HZIF-8-D immobilization synthesis of HZIF-8. However, the XRD pattern suggests a flattening of the Cu NPs' peak strength, indicating a more even distribution.^40^

Fig. 3b and c show the isothermal nitrogen adsorption and desorption curves for HZIF-8, LAC@HZIF-8-D, LAC@HZIF-8-P, and nCu/LAC@HZIF-8. All exhibit a type IV curve with slight uptake at a lower relative pressure (*P*/*P*_0_ < 0.8) and hysteresis loops at a high relative pressure (*P*/*P*_0_ > 0.9).^41^ The BET specific surface area of the LAC@HZIF-8-D-P sample was 1021 m^2^ g^−1^, slightly smaller than that of the LAC@HZIF-8-D sample. Table S1† shows the total surface area alongside the microporous and mesoporous specific surface areas of the ZIF-8 and its immobilized laccase material. The microporous specific surface areas of the LAC@HZIF-8-D and LAC@HZIF-8-P samples were 649 and 610 m^2^ g^−1^, and their mesoporous areas were 468 and 410 m^2^ g^−1^, respectively. The large number of mesopores (Fig. 3d) ranging from 2 to 5 nm corresponded to the internal defects of the crystal during the synthesis of HZIF-8.^42^ The image shows that the 2–5 nm mesopores of the LAC@HZIF-8-D were almost unchanged relative to HZIF-8, while mesopores whose sizes equalled the LAC@HZIF-8-P were sharply reduced. This suggests that the added laccase occupied the mesopores of the HZIF-8, reducing the mesoporous surface area, similar to Gao's findings.^43^ Moreover, the BET specific surface areas of the MZIF-8 and HZIF-8 were 585 and 1153 m^2^ g^−1^, while their mesoporous specific surface areas were 124 and 736 m^2^ g^−1^, respectively (Fig. S3†). Thus, the HZIF-8 had a larger specific surface area, a feature attributable to the addition of histidine and CTAB during its synthesis, which functioned as a stable, common template and facilitated the preparation of pure ZIF-8. Additionally, the N_2_ absorption of nCu/LAC@HZIF-8-D was slightly lower than that of LAC@HZIF-8-D (Fig. 3b). Moreover, as Fig. 3c demonstrates, the micropore-specific surface area of the two changed minimally, while the mesoporous specific surface area of the nCu/LAC@HZIF-8-D significantly decreased.

### 3.2 Development of LAC@HZIF-8 for 2,4-DCP degradation

**Fig. 4:**
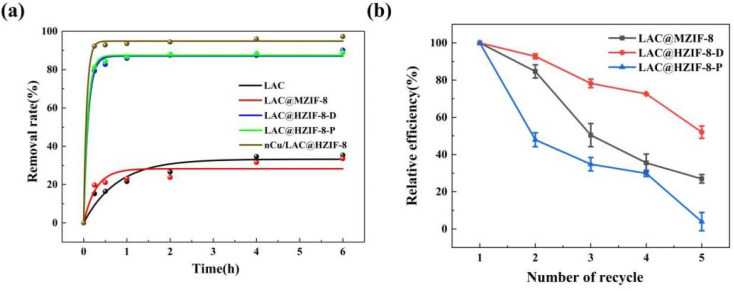
(a) The catalytic removal efficiencies of 2,4-DCP over time. (b) The recycling ability of LAC@ZIF-8 *vs.* free laccase.

Fig. 4a plots the removal efficiencies of 2,4-DCP catalysed by LAC, LAC@MZIF-8, LAC@HZIF-8-D, and LAC@HZIF-8-P over time. The capacity of the LAC@MZIF-8 to remove 2,4-DCP was similar to that of free laccase, with 19.7% and 15.2% removed after 15 min, respectively. However, at the equivalent time, 79.4% and 81.3% of the 2,4-DCP had been removed using LAC@HZIF-8-D and LAC@HZIF-8-P, respectively, indicating the utility of the HZIF-8 immobilized laccase material for removing 2,4-DCP. Two inferences can be drawn from these results. First, in the multistage framework, the laccase is embedded in the mesoporous channels, while the reactants diffuse through microporous channels and reach the active laccase easily. The products can also be quickly separated from the framework, promoting the catalytic reaction.^44–46^ Second, the high surface energy enables the HZIF-8 to accelerate the diffusion of the laccase from solution into catalyst itself by the adsorption to laccase with the high surface energy of structure of HZIF-8.^47^ When the capacities of MZIF-8 and HZIF-8 to adsorb 2,4-DCP were studied to confirm the effect of different material structures on the catalyst, it was observed that 17.6% and 45.4% of 2,4-DCP had been adsorbed into the MZIF-8 and HZIF-8 samples after 15 minutes, respectively. HZIF-8 offers a higher adsorption capacity due to its higher specific surface area, a finding consistent with our findings in this study and those of Gascon.^48^ Overall, HZIF-8 has a higher adsorption capacity and better LAC distribution than MZIF-8. These features explain the catalytic removal capacity of the LAC@HZIF-8 material and indicate the tremendous potential of HZIF-8 as a carrier of enzyme immobilization.

Because the free enzyme is soluble in water and cannot be recycled, reusable composite laccase materials are required for environmental applications. Thus, the reusability of the LAC@ZIF-8 material was tested by successive 2,4-DCP removal cycle experiments. After three catalytic cycles (Fig. 4b), the LAC@HZIF-8-D maintained a 2,4-DCP removal efficiency of about 80%, a higher level of reusability than that of the LAC@HZIF-8-P. This could be attributed to the strong force resulting from the interaction between the biological interface of the enzyme and the metal ions when the LAC@HZIF-8 was prepared *in situ*. This force was much greater than that resulting from pore diffusion and the three-dimensional pore adsorption of laccase in LAC@HZIF-8-P. The excellent reusability of LAC@HZIF-8-D is demonstrated by these results. Some of the adsorbed 2,4-DCP remains in the ZIF-8 pores when the LAC@ZIF-8 is recycled, and leaches into the solution at an increased concentration. The removal efficiency decreases indirectly as the capacity of LAC@ZIF-8 to catalyse 2,4-DCP drops.

When the ability of nCu/LAC@HZIF-8 to catalyse 2,4-DCP in water was evaluated, the rate constant was 1.5 times higher than the best performance achieved by LAC@HZIF-8-D. After 15 min, the 92.3% of the 2,4-DCP had been removed by the nCu/LAC@HZIF-8, which was 12.9% higher than that achieved by LAC@HZIF-8-D. Thus, the Cu nanoparticles significantly improved the material's catalytic performance (Fig. 4a), possibly by increasing its electrical conductivity. This result thereby supports the use of nCu/LAC@HZIF-8 for 2,4-DCP removal. Cu is laccase's active metal centre and its nanoparticles may activate laccase catalysis.^49^ In general, the synthesized nCu/LAC@HZIF-8 composite can remove more than 90% of 2,4-DCP within 15 min. Overall, the degradation of the catalytic activity of enzymes immobilized on MOFs has been a persistent concern which, the current study shows, can be addressed by adding copper nanoparticles.

### 3.3 Stability of LAC@ZIF-8 assessment

**Fig. 5:**
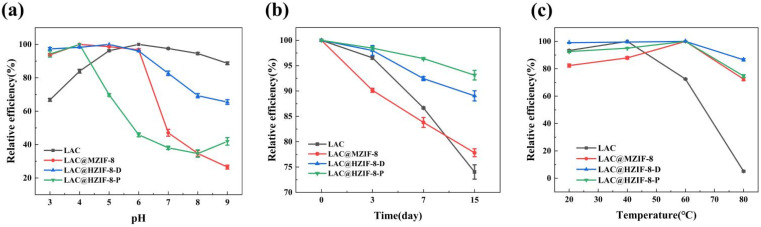
(a) pH stability, (b) thermo-stability, and (c) storage stability of LAC@ZIF-8 compared to free laccase.

Fig. 5a shows the changes over 4 h in the relative activity of the LAC, LAC@MZIF-8, LAC@HZIF-8-D, and LAC@HZIF-8-P samples in different pH buffers (3.0, 4.0, 5.0, 6.0, 7.0, 8.0, and 9.0) in the catalytic removal of 2,4-DCP. The free laccase achieved its optimal catalytic activity at pH 6, reaching 66.8% of this level at pH 3 and 88.7% at pH 9, thereby demonstrating intense catalytic activity under acidic conditions. The optimum catalytic activity of the LAC@MZIF-8 and LAC@HZIF-8 immobilized laccase samples was achieved at pH 4–5, and its catalytic activity was stable under acidic conditions, without any significant decrease. The stability of LAC@ZIF-8 can be attributed to the poor acid stability of ZIF-8 as a sacrificial agent.^50^ However, under neutral and alkaline conditions (pH 7–9), the catalytic activity of the three immobilized laccase samples decreased to varying degrees—and fell significantly under alkaline conditions compared to the free laccase sample. It was speculated that temperature, agitation, ultrasonic interference, and other conditions involved in the process of synthesizing immobilized laccase with ZIF-8 destroyed the enzyme's activity and was manifested in its reduced stability in alkaline pHs.

In another noteworthy result, the catalytic activity of the free laccase decreased by 40% under the pH 3 condition, while the catalytic activity of the LAC@ZIF-8 decreased by less than 5%. This poor activity under a lower pH range may be due to the release of Zn^2+^ as the ZIF-8 skeleton decomposed under acidic conditions, causing the imidazole ligand to bind with H^+^ in solution. This result indicates that a specific pH buffer is required to limit the destruction of the catalytic activity of the LAC@ZIF-8 under acidic conditions. In addition, it has been reported that various metal ions can activate or inhibit the enzyme activity of laccase.^51^ Among these, Co^2+^, Ni^2+^, Cd^2+^, Fe^3+^, Ag^+^, Al^3+^, Hg^2+^, and Fe^2+^ were strong inhibitors, possibly because they occupied the laccase's active centre and changed its structure, thus inhibiting its enzymatic activity.^52,53^ However, Na^+^, Zn^2+^, Cu^2+^, and Mg^2+^ ions can significantly activate laccase. The activation effect of Cu^2+^ on laccase is related to the fact that Cu^2+^ is an active centre of laccase.^54^ Therefore, the enhanced stability of the LAC@ZIF-8 materials under acidic conditions may be due to the Zn^2+^ produced by the instability of the ZIF-8 structure, which enhances the enzyme activity of laccase, to a certain extent.

Fig. 5b shows the relative activity changes in 2,4-DCP removal achieved by the LAC, LAC@MZIF-8, LAC@HZIF-8-D, and LAC@HZIF-8-P samples treated at different temperatures (20, 40, 60, and 80 °C) for 4 h. The catalytic activity of the free laccase was optimal at 40 °C, decreasing to 93% of this value at 20 °C. However, at increased temperatures, its catalytic activity was reduced significantly. At 80 °C, the catalytic activity of the laccase dropped to 5.1%, meaning it was almost wholly inactivated, a finding consistent with Wu *et al.*^55^ High temperatures destabilize free laccase, causing a loss of activity due to the decomposition of its protein structure. In contrast, the immobilized laccase material was stable at a range of temperatures; the optimum temperature was 60 °C, with higher catalytic activity observed between approximately 20 and 80 °C. At 80 °C, the catalytic activity of the LAC@MZIF-8 and LAC@HZIF-8 was maintained at 72.4 and 86.5% of this value respectively, indicating that the ZIF-8 greatly enhanced laccase durability at high temperatures. These results corroborate earlier research showing that the optimal temperature for immobilized laccase increased as catalytical activation energy rose due to the chelation between the laccase and ZIF-8.^56^ Moreover, the HZIF-8 demonstrated greater temperature stability than the MZIF-8, a finding related to the latter's poor crystallinity and pore properties.

Finally, Fig. 5c shows the changes in relative activity among the LAC, LAC@MZIF-8, LAC@HZIF-8-D, and LAC@HZIF-8-P samples after storage at 4 °C for 0, 3, 7, and 15 days. After 15 days of storage at 4 °C, the catalytic activities of both the free and immobilized enzyme samples did not decrease significantly, indicating that laccase has a long storage cycle in optimal storage environments at 4 °C. However, the immobilized laccase material was more stable than the free laccase, since HZIF-8 retains the catalytic activity from LAC and has greater LAC storage capacity due to its hierarchical pore structure.

## 4 Conclusions

This study investigated the ability of HZIF-8 to immobilize laccase for the removal of 2,4-DCP, a widespread pollutant. The multistage structure of HZIF-8 enabled LAC@HZIF-8 to remove approximately 80% of 2,4-DCP under optimal conditions—about 5 times the removal efficiency of LAC@MZIF-8. In addition, LAC@HZIF-8 also enhanced the thermostability and storage stability of laccase compared to LAC@MZIF-8. Due to the size mismatch between laccase and the material's micropores, post-synthesis embedded laccase at a higher rate than the *de novo* strategy. However, the latter produced a higher removal efficiency when Cu NPs were added and can potentially broaden the pH stability of laccase. In conclusion, the LAC@HZIF-8-D showed greater promise for environmental applications due to its higher removal efficiency and excellent stability. The design of graded MOF channels and the pore size of mesoporous channels based on the molecular size of enzymes were also significant in enzyme immobilization.

## Author contributions

Xu Ran and Zhang Xujie performed and conducted the experiments, Osamn Ahmend drafted the manuscript and analyzed the data, and Wu Yi-nan and Eduardo Schott supervised the study. All authors were involved in manuscript editing.

## Conflicts of interest

There are no conflicts to declare.

## Supplementary Material

RA-013-D3RA01571H-s001
