# Supplementary material for: Improved stability and activity of laccase through de novo and post-synthesis immobilization on a hierarchically porous metal–organic framework (ZIF-8)
Source: RSC Adv. 2023 Jun 8;13(25):17194–201. doi: 10.1039/d3ra01571h (PMC10248541; doi:10.1039/d3ra01571h)
Supplement: RA-013-D3RA01571H-s001 [file RA-013-D3RA01571H-s001.pdf]

Electronic Supplementary Material (ESI) for RSC Advances.
This journal is © The Royal Society of Chemistry 2023

Supporting Information for

Improved stability and activity of laccase through de novo and post-synthesis immobilization on a hierarchically porous metal-organic framework (ZIF-8)

Ran Xu,[ab] Xujie Zhang, [ab] Osman Ahmend Zelekew[abc] , Eduardo Schott[*d] and Yi-nan Wu[*ab]

a.College of Environmental Science and Engineering, State Key Laboratory of Pollution Control and Resource Reuse, Tongji University, 1239 Siping Rd., Shanghai 200092, China

b.Shanghai Institute of Pollution Control and Ecological Security, 1239 Siping Rd., Shanghai 200092, China

c.Department of Materials Science and Engineering, Adama Science and Technology University, Adama, Ethiopia

d. Department of Inorganic Chemistry of the Faculty of Chemistry and Pharmacy, Pontificia Universidad Católica de Chile, Vicuña Mackenna 4860, Macul, Santiago, Chile

[*] Corresponding author. Email address: 51n@tongji.edu.cn, edschott@uc.cl

Synthesis of copper (Cu) nanoparticles

A 0.19 g of sodium borohydride was dissolved in deionized water followed by adding sodium hydroxide solution (1 mol/L) and assigned as solution A with pH 12. Then, copper sulfate pentahydrate (2.50 g) was dissolved into 100 mL of anhydrous ethanol and water mixture solution (v/v, 2: 3) and the appropriate amount of polyethylene pyrrolid1 (PVP) was added (solution B). Then, solution A was added drop by drop into solution B under stirring for 1 h. The product was then washed by water three times and dried overnight in a vacuum drying oven at 80°C.

Synthesis of MZIF-8 and LAC@MZIF-8

To synthesize MZIF-8, $Zn(NO_3)_2 \cdot 6H_2O$ (1 mmol) was dissolved in deionized water (25 mL). Subsequently, 2-methylimidazole (8 mmol) was dissolved in deionized water (25 mL), and triethylamine (8 mmol) was added to the solution. The $Zn(NO_3)_2 \cdot 6H_2O$ and 2-methylimidazole solutions were then mixed and placed on a magnetic stirrer for 1 h at room temperature. After centrifugation and freeze-drying overnight, the white MZIF-8 powder was obtained.

The de novo synthesis of the LAC@MZIF-8 MOF was performed as follows: First, a certain quantity of laccase was dissolved in a pH 5 citric acid - disodium phosphate (CPBS) buffer solution (1 mL). This was then added to a $Zn(NO_3)_2 \cdot 6H_2O$ solution under ultrasonication, with the procedure then following that of the MZIF-8 preparation process described above.

Catalytic curve fitting method

The first-order reaction kinetics equation (Equation S1) was used to perform the nonlinear fitting for the removal rate of 2, 4-DCP catalyzed by laccase, LAC@MZIF-8, and LAC@HZIF-8 samples. According to the fitting parameter R^2, the time kinetics of the catalytic removal of 2, 4-DCP by laccase and ZIF-8 immobilized laccase material was in line with the first-order reaction kinetics equation. The catalytic removal rate had an exp1ntial correlation with time(t). The catalytic rate constant per hour of LAC@HZIF-8 was the highest, indicating that the catalytic efficiency of 2, 4-DCP immobilized by HZIF-8 was the best.

$$q_t = q_e(1 - e^{-k_1 t}) \quad (1)$$

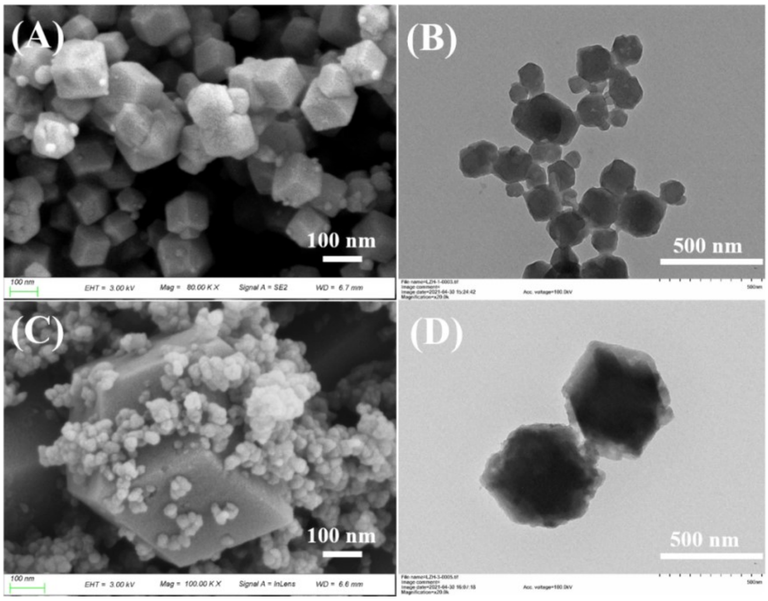

Fig S1. SEM&TEM patterns of MZIF-8, and LAC@MZIF-8. (a) SEM image of MZIF-8; (b) TEM image of MZIF-8; (c) SEM image of LAC@MZIF-8; (d) TEM image of LAC@MZIF-8

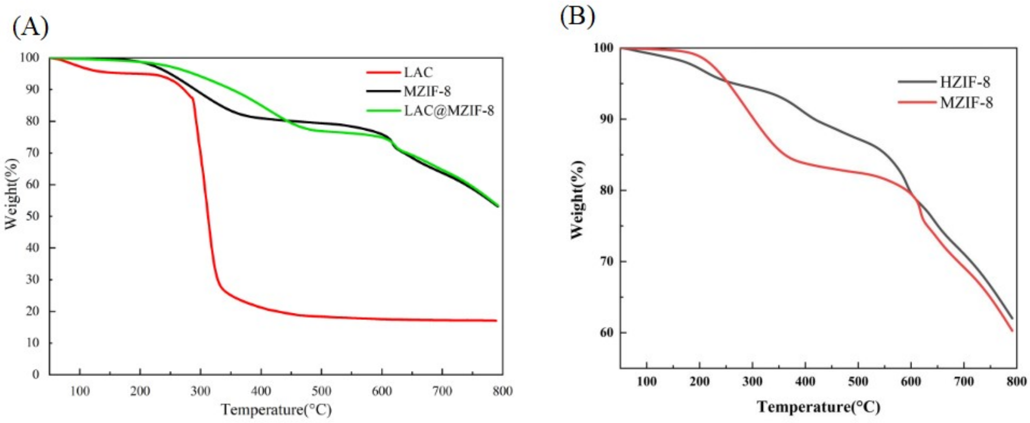

Fig S2. The TGA curve of (a) LAC, MZIF-8，LAC@MZIF-8; (b) MZIF-8, HZIF-8

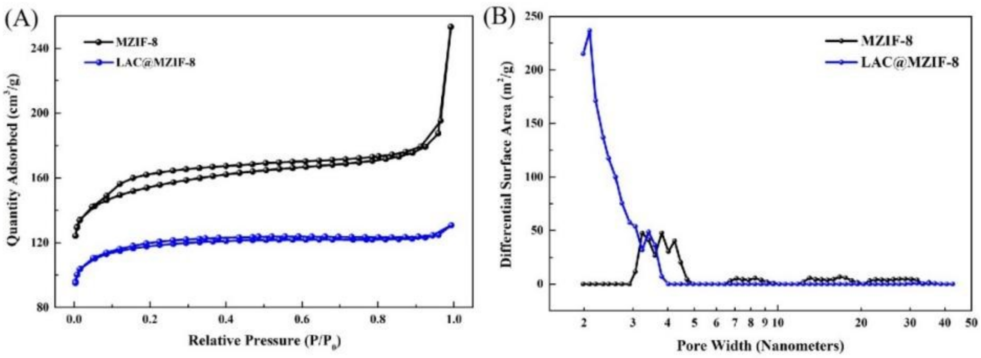

FigS3. N_2 adsorption/desorption isotherms and the pore size distribution images of MZIF-8 and LAC@MZIF-8. (a) N_2 adsorption/desorption isotherms image of the MZIF-8, and LAC@MZIF-8; (b) pore size distribution image of

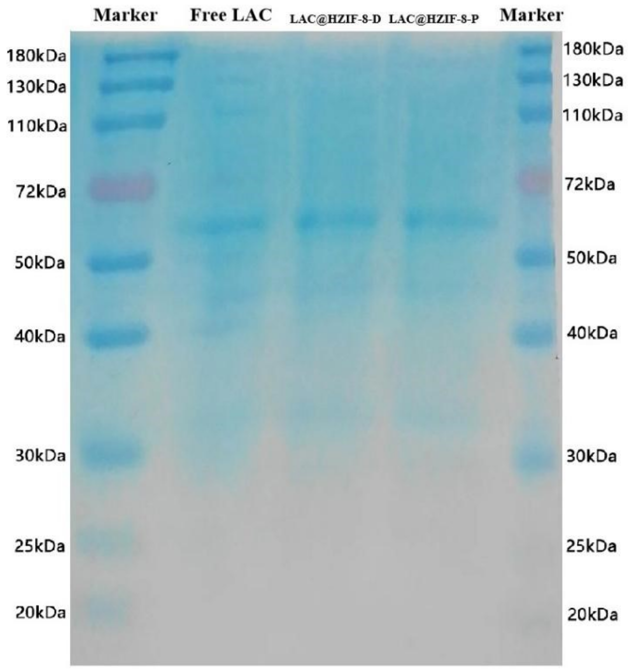

the MZIF-8, and LAC@MZIF-8.

Fig S4. Electrophoresis gel of (from the left to the right): (a) a protein Marker; (b) Free LAC; (c) LAC@HZIF-8-D and (d) LAC@HZIF-8-P.

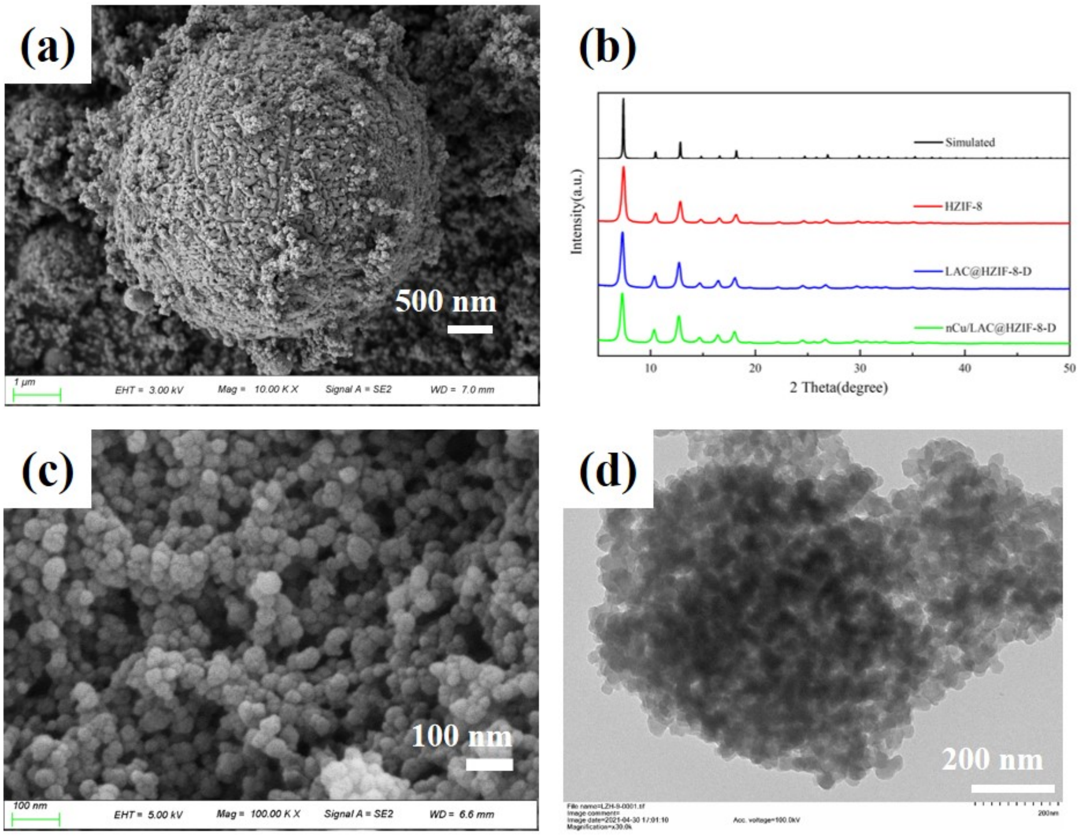

Fig S5. (a) SEM pattern of the nCu; (b) The XRD pattern of HZIF-8, LAC@HZIF-8-D, nCu/LAC@HZIF-8-D ; (c) SEM pattern of the nCu/LAC@HZIF-8; (d) TEM pattern of the nCu/LAC@HZIF-8

TableS1. The specific surface area of ZIF-8, LAC@ZIF-8-D and LAC@HZIF-8-P

Sample	S_{BET} (m^2/g)	S_{micro} (m^2/g)	S_{meso} (m^2/g)
MZIF-8	585	460	124
LAC@MZIF-8	452	387	65
HZIF-8	1153	736	417
LAC@HZIF-8-D	1118	649	468
LAC@HZIF-8-P	1021	610	410
